# Curcumin Reverses Antibiotic Resistance and Downregulates Shiga Toxin Expression in Enterohemorrhagic *E. coli*

**DOI:** 10.3390/diseases13050154

**Published:** 2025-05-17

**Authors:** Martin Zermeño-Ruiz, Mirian Cobos-Vargas, Mauro Donaldo Saucedo-Plascencia, Rafael Cortés-Zárate, Leonardo Hernandez-Hernandez, Teresa Arcelia Garcia-Cobian, Teresa Estrada-Garcia, Araceli Castillo-Romero

**Affiliations:** 1 Departamento de Farmacobiología, Centro Universitario de Ciencias Exactas e Ingenierías, Universidad de Guadalajara, Blvd. Marcelino García Barragán #1421, Guadalajara 44430, Mexico; martin.zermeno@academicos.udg.mx; 2 Departamento de Microbiología y Patología, Centro Universitario de Ciencias de la Salud, Universidad de Guadalajara, Calle Sierra Mojada 950, Independencia Oriente, Guadalajara 44340, Mexico; mirian.cobos4584@alumnos.udg.mx (M.C.-V.); rafael.czarate@academicos.udg.mx (R.C.-Z.); 3 Departamento de Fisiología, Centro Universitario de Ciencias de la Salud, Universidad de Guadalajara, Calle Sierra Mojada 950, Independencia Oriente, Guadalajara 44340, Mexico; mauro.saucedo3612@alumnos.udg.mx (M.D.S.-P.); leonardo.hernandez@cucs.udg.mx (L.H.-H.); arcelia.garcia@academicos.udg.mx (T.A.G.-C.); 4 Departamento de Biomedicina Molecular, Centro de Investigación y Estudios Avanzados del Instituto Politécnico Nacional, Av. Instituto Politécnico Nacional 2508, Col. San Pedro Zacatenco, Alcaldía Gustavo A. Madero, Ciudad de México 07360, Mexico; testrada@cinvestav.mx

**Keywords:** enterohemorrhagic *Escherichia coli*, curcumin, antimicrobial resistance

## Abstract

Background: Enterohemorrhagic *Escherichia coli* (EHEC) is a considerable public health concern associated with several foodborne outbreaks of bloody diarrhea (BD) and the potentially lethal hemolytic uremic syndrome (HUS), the pathophysiology of which is attributable to the Shiga toxin (Stx) produced by this bacterium. In most patients, supportive treatment will be sufficient; however, in some cases, antibiotic treatment may be necessary. Most antibiotics are not recommended for EHEC infection treatment, particularly those that kill the bacteria, since this triggers the release of Stx in the body, inducing or worsening HUS. Azithromycin, which prevents the release of Stx and is a weaker inducer of the SOS system, has been successfully used to reduce EHEC shedding. It is necessary to identify compounds that eliminate EHEC without inducing Stx release. The use of natural compounds such as curcumin (CUR), a polyphenol derived from turmeric, has been highlighted as an alternative bactericidal treatment approach. Objective: The objective of this study was to establish the effect of CUR and its interactions with selected antibiotics on resistant EHEC O157/H7/EDL933. Methods: Bacterial cultures were exposed to CUR at three different concentrations (110, 220, and 330 µg/mL) and 1.2% DMSO, and the antimicrobial activity of CUR was assessed by measuring the optical density at 600 nm (OD600). The synergy of CUR and the antibiotics was determined with the FIC method. RT-PCR was performed to determine the expression levels of the *bla_CTX-M-15_*, *catA1*, *acrAB-tolC stx2A*, and *stx2B* genes. Results: Our data indicate that CUR did not affect the growth of EHEC, but when combined with the antibiotics, it acted as a bacterial resistance breaker. Synergistic combinations of CUR and cefotaxime or chloramphenicol significantly reduced colony counts. Conclusions: Our findings support the potential of CUR as a sensitizer or in combination therapy against EHEC.

## 1. Introduction

Pathogenic variants of Enterohemorrhagic *E. coli* (EHEC) are responsible for causing hemorrhagic gastroenteritis, which can progress to hemolytic uremic syndrome (HUS), a critically important public health concern characterized by acute renal failure, hemolytic anemia, and thrombocytopenia, with a mortality rate ranging from 5 to 10%, particularly in young children and immunocompromised individuals in the absence of careful clinical management [1,2,3].

The development of HUS is associated with the production and secretion of a potent toxin, Stx (Shiga-like toxin), which is responsible for severe disease. The authors of several studies have discussed the relationship between conventional antibiotics and exacerbated Stx production, as well as increased risk of HUS [4,5,6,7]. Due to the risk of triggering HUS, for most patients, supportive treatment will be sufficient for EHEC treatment. However, in some cases, such as when bacteremia occurs or to reduce environmental shedding, antibiotic treatment may be necessary. Macrolides such as azithromycin that do not induce Stx production have been proposed in some regions. However, there are still many questions about azithromycin eradication therapy. In addition, the growing issue of EHEC macrolide resistance, which leads to treatment failure and complications, is a cause for urgent concern in the field of infectious diseases [8]. New aspects regarding several non-Stx-inducing antibiotics in EHEC infections and HUS have been investigated. Cefotaxime and chloramphenicol, which exert their bactericidal actions by inhibiting the synthesis of the bacterial cell wall and proteins, respectively, do not affect Stx gene expression and decrease the phage and Stx2 titers, providing a positive outlook and making these options attractive [9]. In *E. coli* O157:H7 in vitro culture, cefotaxime had no significant inductive effects on *stx2* expression, but chloramphenicol strongly increased stx2 gene expression and its release into the culture supernatant [6]. During an EHEC O104:H4 outbreak, a diverse panel of antibiotics were tested, and their role in the induction of Stx2 was evaluated; in the isolates, chloramphenicol significantly decreased stx2 transcription [9]. These findings underscore the potential of these antibiotics as promising treatment options against EHEC but also highlight the urgent need for further research in this area.

On the other hand, several studies have demonstrated the antibacterial activity of phytochemicals, including flavonoids, terpenoids, alkaloids, and phenolic compounds. In EHEC, phytochemicals are known to disrupt biofilm formation and affect cell motility and gene expression; interestingly, they downregulate Shiga toxin gene expression [10,11]. The natural phenol curcumin (CUR) [1,7-bis (4-hydroxy-3-methoxyphenyl)-1,6 heptadiene-3,5-dione] is the primary curcuminoid of turmeric (*Curcuma longa* L). CUR has been recognized as a safe molecule and has been used for centuries as a spice and in traditional Chinese and Indian medicine; it is known for its potential, for example, as a bactericidal [12]. In food systems, CUR treatment combined with UV-A light was able to inactivate *E. coli* O157:H7 [13]. Other studies have demonstrated that CUR resensitizes resistant bacteria. The synergistic activity of CUR with fluoroquinolones, beta-lactams, and aminoglycosides reduces the minimum inhibitory concentration (MIC) of antibiotics, thereby enhancing their antimicrobial activity [14,15]. Furthermore, a combination of CUR with antibiotics has been associated with cell membrane disruption, reduced efflux pump activity, and decreased levels of proteins involved in bacterial resistance [16].

The antibacterial activity of CUR has been the subject of extensive research; however, definitive conclusions about its therapeutic potential in *E. coli* pathotypes, particularly EHEC, remain limited. With this study, we aim to fill this gap by exploring the efficacy of CUR alone and its synergistic effects when combined with cefotaxime or chloramphenicol against multidrug-resistant EHEC.

## 2. Materials and Methods

### 2.1. Bacterial Strain

A strain of enterohemorrhagic Escherichia coli (EHEC) serotype O157:H7, namely, EDL933, was kindly provided by Dr. Estrada-García of the Research Centre and Advanced Studies of the National Polytechnic Institute (CINVESTAV, Ciudad de México, Mexico).

### 2.2. Maintenance and Preservation of Microorganisms

The bacterial strain was grown on nutrient agar plates (Becton, Dickinson and Company, Franklin Lakes, NJ, USA) at 37 °C for 18–20 h. An isolated colony was streaked onto a fresh agar plate every seven days to maintain bacterial cultures. Stock cultures were stored in Luria–Bertani (LB) broth (Sigma-Aldrich, St. Louis, MO, USA) containing 25% glycerol at −80 °C.

### 2.3. CUR

CUR was purchased from Sigma-Aldrich, MO, USA (≥65% purity; HPLC) and was dissolved in dimethyl sulfoxide (DMSO; Sigma-Aldrich, MO, USA) at a 40 mg/mL final concentration. For the killing assays, the working concentrations (110, 220, and 330 µg/mL) were diluted in phosphate-buffered saline (PBS) at pH 7.2, such that the DMSO concentration remained 1.2%.

### 2.4. Antibacterial Activity of CUR

The antibacterial activity of CUR was evaluated by growing the bacterial strain in LB broth (Sigma-Aldrich, MO, USA) at 37 °C and 250 rpm. The cultures were allowed to grow until they reached an optical density (OD600) of 0.08 (~10^7^ colony-forming units (CFU)/mL). Subsequently, the cells were pelleted by centrifugation at 1844× *g* for 5 min (Microcentrifuge Sigma 1-14K, Sigma Laborzentrifugen GmbH, Osterode am Harz Germany) and then resuspended in 3 mL of PBS containing CUR at 110, 220, or 330 µg/mL. The cells were incubated for 2 h at 37 °C and 250 rpm. Untreated and 1.2% DMSO-treated cultures were included as negative controls. After the incubation period, the cells were harvested through centrifugation and washed twice with sterile PBS to remove excess CUR. They were then resuspended in sterile PBS to create eight serial dilutions (1:10) of each treatment, with a final volume of 3 mL. Subsequently, 100 µL of each dilution was inoculated into 20 mL of molten nutrient agar (Sigma-Aldrich, MO, USA), cooled to 55 °C, gently mixed, and poured into sterile 90 mm diameter Petri dishes. The plates were incubated overnight at 37 °C. Bacterial growth was calculated based on CFU counting. The dilutions chosen produced between 30 and 300 separate countable colonies. All experiments were performed in triplicate.

### 2.5. EHEC Resistance Assay

The resistance or susceptibility of EHEC to cefotaxime and chloramphenicol was evaluated with the Kirby–Bauer disk diffusion method [17]. Briefly, an overnight culture in LB broth (Sigma-Aldrich, MO, USA) was diluted 100 times with Mueller–Hinton broth to a final volume of 3 mL and incubated at 37 °C and 250 rpm. The growth was monitored until the absorbance at 600 nm reached about 0.4 (~3.2 × 10^8^ cells/mL). The suspension was then used to inoculate Mueller–Hinton agar plates by swabbing. The inoculated plates were allowed to dry for five minutes, and filter paper disks (BD BBL Sensi-Disc, Becton, Dickinson and Company, NJ, USA) containing the test compounds at a known concentration were placed on the agar surfaces. The plates were incubated at 35 ± 2 °C for 20 h [18,19]. After this time, the diameter of the growth inhibition zone surrounding the antibiotic disks was measured with calipers. The reading of the inhibition zones was interpreted by using the criteria published by the Clinical and Laboratory Standards Institute [17].

### 2.6. Determination of Minimum Inhibitory Concentration

The susceptibility to cefotaxime and chloramphenicol (Sigma-Aldrich, MO, USA) was quantified with the microdilution technique [20]. An overnight EHEC culture was diluted 100 times in 3 mL of Mueller–Hinton broth and then incubated at 37 °C and 250 rpm until the absorbance at 600 nm reached about 0.01 (~8.0 × 10^6^ CFU/mL). Subsequently, 100 µL of bacterial inoculum was mixed with 100 µL of serial dilutions of antibiotics (cefotaxime, 3.84 µg/mL to 0.075 µg/mL, and chloramphenicol, 256 µg/mL to 0.5 µg/mL) in 96-well plates. The plates were incubated at 37 °C for 18 h. Wells containing only Mueller–Hinton broth or broth in conjunction with a bacterial suspension were used as sterility and negative control experiments, respectively. Bacterial wells treated only with 1.2% DMSO served as the diluent controls. The optical density (OD) at a wavelength of 600 nm was measured by using a Multiskan FC plate reader (Thermo Fisher Scientific Inc., Rockford, IL, USA). The MIC was determined as the lowest antibiotic concentration that resulted in the complete eradication of EHEC compared with the control.

### 2.7. Sensitizing and Synergistic Antibacterial Effects of CUR and Antibiotics

We next assessed the potential of CUR to enhance the susceptibility of EHEC to antibiotics. The strain was exposed to DMSO or 110, 220, or 330 µg/mL CUR for 2 h, following the previously described procedure. Afterwards, the cells were harvested by centrifugation and resuspended in 3 mL of PBS to an optical density (OD) at 600 nm of approximately 0.4 (approximately 3.2 × 10^8^ cells/mL). The susceptibility of the sensitized EHEC to cefotaxime or chloramphenicol was analyzed by employing the Kirby–Bauer disk diffusion method.

To evaluate whether CUR had a synergistic effect with cefotaxime and chloramphenicol, both the agar disk diffusion and broth dilution methods were used. The diffusion method involved Mueller–Hinton agar plates supplemented with 110, 220, or 330 µg/mL CUR. The inoculum, inoculation on MH agar–CUR plates, and antibiotic susceptibility testing were performed according to the previously mentioned procedure. The inhibition zones in the presence of CUR were measured and compared to the controls.

For the broth dilution assay, CUR was combined with cefotaxime (0.24 μg/mL) or chloramphenicol (64 μg/mL). Overnight EHEC cultures were diluted 100-fold in Mueller–Hinton broth to a final volume of 3 mL and incubated at 37 °C with shaking at 250 rpm until the OD600 reached 0.1 (8.0 × 10^7^ cells/mL). Subsequently, combinations of CUR and either cefotaxime or chloramphenicol were introduced, and the cultures were then incubated at 37 °C for 18 h. The assessment of bacterial growth was conducted by counting the CFU after plating 100 µL of a 10^−7^ dilution onto Mueller–Hinton agar plates. The synergy was determined by using the Fractional Inhibitory Concentration (FIC) method, as follows: FIC = (CFU_AB_/CFU_A_) + (CFU_AB_/CFU_B_), where CFU_AB_ is the treatment combination of CUR with one of the antibiotics, CFU_A_ is the treatment with CUR, and CFU_B_ is the treatment with one of the antibiotics.

The following interpretation was adopted: an FICI ≤ 0.5 indicates synergistic interaction, an FICI between 0.5 and 1 indicates an additive effect, an FICI between 1 and 4 indicates an indifferent effect, and an FICI > 4 indicates an antagonistic effect [21]. Each experiment was performed in triplicate and repeated thrice.

### 2.8. Effect of Curcumin on Gene Expression of Resistant EHEC

RNA from DMSO and CUR-exposed EHEC was extracted by using the Total RNA Purification Kit (NORGEN) as recommended by the manufacturer. RNA samples were quantified by directly measuring the absorbance at 260 nm with a BioPhotometer D30 (Eppendorf SE, Hamburg, Germany). The absence of DNA contamination and RNA integrity were analyzed on 1.0% agarose gel [22]. The first strand of cDNA was synthesized with a reverse transcriptase reaction by using the commercial Verso cDNA Synthesis Kit (Thermo Fisher Scientific Inc., Rockford, IL, USA) with 1 µg of RNA. RT-PCR primers were designed by using the following sequences from GenBank: *acrA* (GenBank Accession MT956577.1), *acrB* (GenBank Accession NZ_BJPK01000236.1), *tolC* (GenBank Accession HQ833339.1), *bla_CTX-M-15_* (GenBank Accession KF723591.1), and *catA1* (GenBank Accession OK206419.1). The expression levels of the mentioned genes were normalized to that of the glyceraldehyde 3-phosphate dehydrogenase gene (*gapdh*) (GenBank Accession X02662.1). Relative quantity RT-PCR was performed in a MiniAmp Thermal Cycler (Thermo Fisher Scientific Inc., Rockford, IL, USA), with 500 ng of cDNA and DreamTaq Green PCR (Thermo Fisher Scientific Inc., Rockford, IL, USA). The PCR primers and amplification conditions are shown in Table 1. The resulting amplicons were visualized by performing electrophoresis on 1% agarose gel. The relative expression was determined with a densitometric assay by using Image Studio Digits Ver 5.2 software.

### 2.9. Effect of CUR on Shiga Toxin Expression

The evaluation of Shiga toxin expression was conducted based on RT-PCR. The EHEC strain was cultivated overnight, after which 25 mL of LB broth was inoculated to an OD600 of approximately 0.4 (~3.2 × 10^8^ cells/mL). Thereafter, 3 mL of the culture was distributed into separate tubes and exposed to 1.2% DMSO, 220 μg/mL CUR, chloramphenicol at sub-MIC (64 μg/mL), cefotaxime at sub-MIC (0.24 μg/mL), chloramphenicol at sub-MIC plus 220 μg/mL CUR, and cefotaxime at sub-MIC plus 220 μg/mL CUR. The experiment was conducted over a period of two hours. Following treatment, RNA extraction was performed as outlined in Section 2.7. The primers for RT-PCR were designed by using the following GenBank sequence: stx2AB (GenBank Accession No. AB052227.1). The PCR primers and amplification conditions are shown in Table 1. As outlined in Section 2.7, the processes of visualization and analysis were conducted in accordance with the specified protocol. The fold change (FC) was determined with the following formula: FC = 2^log2FC, where log2FC = log2(B) − log2(A), B is the combinatorial treatment of CUR with the antibiotics, and A is the treatment with the antibiotics [23].

### 2.10. Statistical Analysis

All data are presented as mean values with standard deviations and were analyzed by using two-way ANOVA, followed by Dunnett’s multiple comparisons test (GraphPad Prism version 6.01 for Windows, GraphPad Software, La Jolla, CA, USA). A *p*-value of ≤0.05 was considered statistically significant.

## 3. Results

### 3.1. CUR Did Not Show Antibacterial Effect

**Figure 1:**
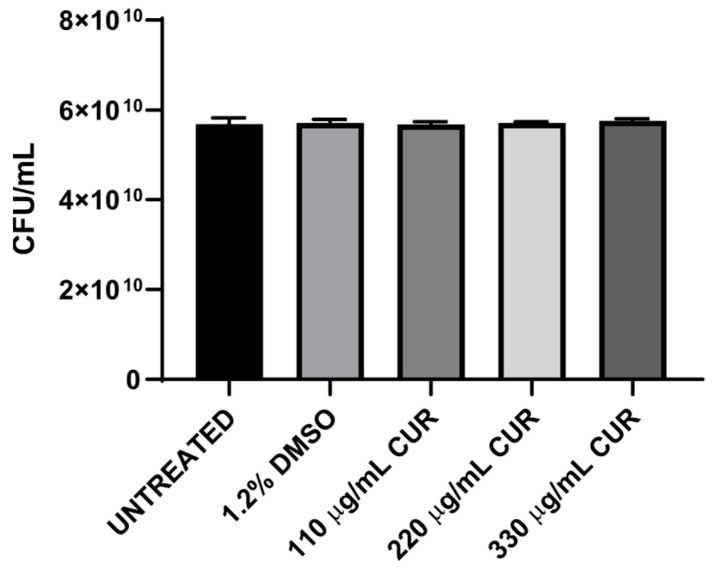
Effect of CUR on growth (CFU) of EHEC.

A concentration of 10^7^ CFU/mL was exposed to DMSO or CUR for 2 h, and the effects were analyzed by using the pour-plate method to assess whether CUR exhibits bactericidal activity against EHEC. The results demonstrate that 110, 220, and 330 μg/mL CUR did not affect the growth of EHEC; the number of recoverable CFU from CUR-treated cultures was not significantly different from that of untreated and DMSO-treated cultures (Figure 1).

### 3.2. Resistance Profile and Minimum Inhibitory Concentration (MIC) of Enterohemorrhagic E. coli

**Figure 2:**
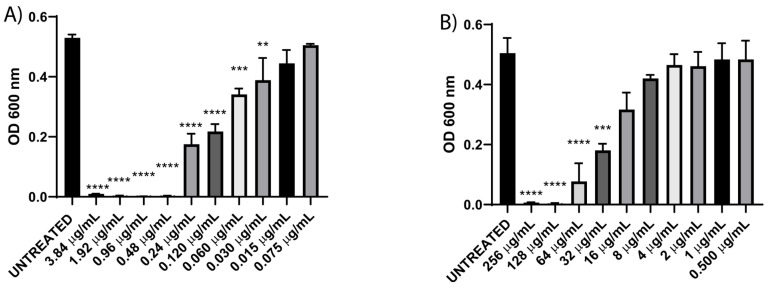
Minimum inhibitory concentration against EHEC of cefotaxime (**A**) and chloramphenicol (**B**). Data correspond to mean values ± SD of three independent experiments carried out in triplicate. ** *p* < 0.01, *** *p* < 0.001, and **** *p* < 0.0001.

EHEC displayed acquired intermedia resistance towards cefotaxime and complete resistance to chloramphenicol (Table 2). When MIC was assessed by using a dilution method, the findings demonstrated that 0.48 µg/mL was the lowest concentration of cefotaxime required to completely inhibit the growth of EHEC (Figure 2A). This result aligns with the clinical breakpoint for cefotaxime sensitivity, which is ≤1 µg/mL [17]. For chloramphenicol, an MIC of 128 µg/mL was observed, indicating resistance to this antibiotic, as per the clinical sensitivity breakpoint of ≤8 µg/mL (Figure 2). These findings corroborate the EHEC strain’s resistance to chloramphenicol.

### 3.3. CUR Sensitizes EHEC to Different Antibiotics

In mammalian cells, the sensitizing effect of CUR against various chemotherapies is well documented [24,25]. Given that EHEC exhibited resistance to cefotaxime and chloramphenicol and that these antibiotics do not induce Stx production, the aim was to employ CUR to increase susceptibility in EHEC. Therefore, we sensitized EHEC with CUR for 2 h before performing an antibiogram assay. Table 3 shows the inhibition zones (in mm). The diameter of the inhibition zone around the disks of cefotaxime and chloramphenicol gradually increased with the increase in the concentration of CUR, making the antibiotic-resistant EHEC susceptible. No differences were observed in untreated and DMSO-treated cultures.

### 3.4. CUR Affects the Relative Expression of Antimicrobial Resistance Genes

**Figure 3:**
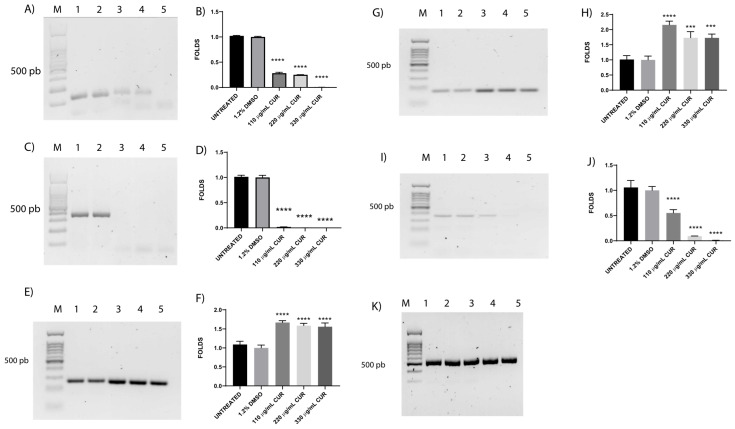
Effects of CUR on relative expression of antibiotic resistance genes after 2 h of treatment. Agarose gels showing expression of *bla_CTX-M-15_* (**A**), *catA1* (**C**), *tolC* (**E**), *acrA* (**G**), *acrB* (**I**), and *gapdh* (**K**). Histograms represent fold changes in densitometry for *bla_CTX-M-15_* (**B**), *catA1* (**D**), *tolC* (**F**), *acrA* (**H**), and *acrB* (**J**) expression levels, as determined with agarose gel analysis and normalized by using *gapdh*. Average values were normalized to *gapdh*. Lane M, 100 bp weight marker; lane 1, untreated; lane 2, DMSO; lane 3, 110 µg/mL CUR; lane 4, 220 µg/mL CUR; lane 5, 330 µg/mL CUR. Data corresponds to mean values ± SD of three independent experiments carried out in triplicate. *** *p* < 0.001, and **** *p* < 0.0001.

Following the changes in antibiotic susceptibility, we investigated the potential effect of CUR on the expression of drug resistance genes. EHEC exposed to CUR, as expected, presents significant downregulation of *bla_CTX-M-15_*, and *acrB*, by 0.004 and 0.007 times, respectively (Figure 3A,I); *catA1* expression with CUR at all doses was almost undetectable (Figure 3C). Interestingly, *tolC* and *acrA* were found to be upregulated, by 1.6 and 1.8 times, respectively, in response to CUR exposure (Figure 3E,G) compared with untreated and DMSO-treated bacteria.

### 3.5. CUR Broke Antibiotic Resistance of EHEC

**Figure 4:**
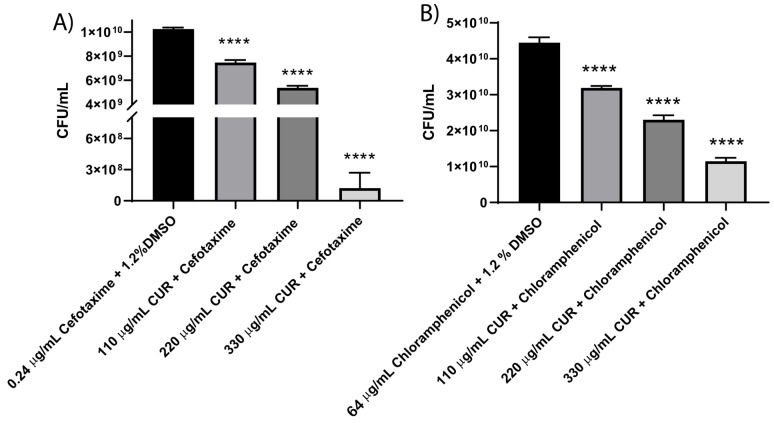
Effect of combinatorial treatment of CUR with cefotaxime (**A**) or chloramphenicol (**B**) on growth (CFU) after 18 h of incubation. Data correspond to mean values ± SD of three independent experiments carried out in triplicate. **** *p* < 0.0001.

It has been reported that combining CUR and cephalosporin has synergistic or additive effects [26]. Previously, we demonstrated the synergistic antibacterial action of CUR with antibiotics against Enterotoxigenic *E. coli* [15]. The results in Table 4 reveal that CUR at all concentrations combined with cefotaxime or chloramphenicol reversed the resistance pattern of EHEC. Additionally, the CFU/mL counts demonstrated a concentration-dependent effect of CUR combined with antibiotics after 18 h of incubation (Figure 4). We used the value of the FIC index as a predictor of synergism, and it was found that only combinations of 330 µg/mL CUR with cefotaxime (0.24 μg/mL) or chloramphenicol (64 μg/mL) exhibited synergistic activity, with FIC indexes of 0.01 and 0.46, respectively. At 110 µg/mL and 220 µg/mL, only an additive effect was observed (Table 5).

### 3.6. Shiga Toxin Is Downregulated by CUR

**Figure 5:**
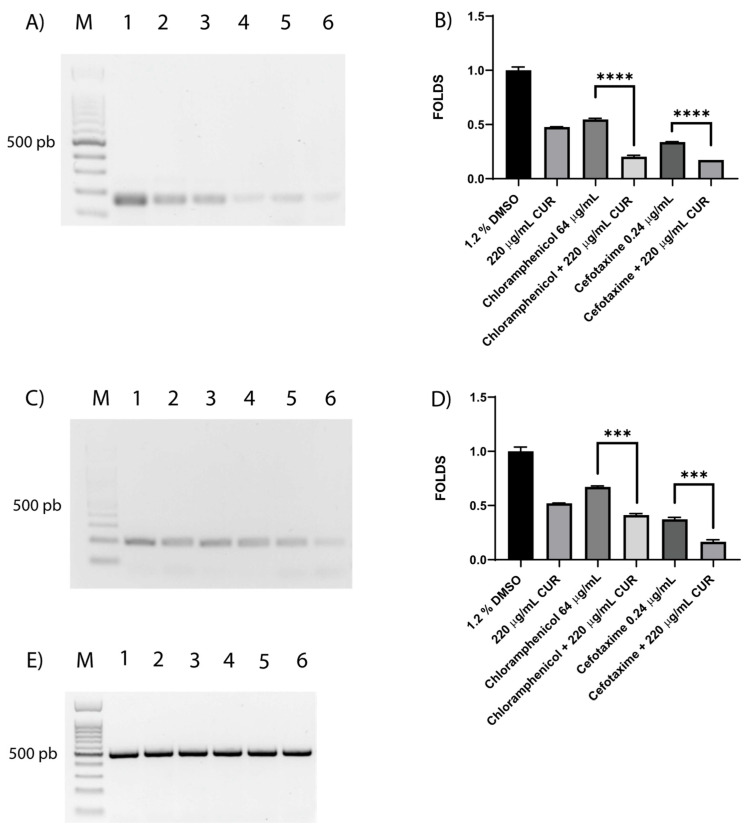
**Effects of CUR, CUR–cefotaxime, and CUR–chloramphenicol on STX gene expression**. Agarose gel images of *stx2a* (**A**), *stx2B* (**C**), and *gapdh* (**E**). Histograms represent fold changes in densitometry for *stx2A* (**B**) and *stx2B* (**D**) expression levels, as determined with agarose gel analysis and normalized by using *gapdh*. Lane M, 100 bp weight marker; lane 1, DMSO; lane 2, 220 µg/mL CUR; lane 3, chloramphenicol 64 µg/mL; lane 4, chloramphenicol 64 µg/mL + 220 µg/mL CUR; lane 5, cefotaxime 0.24 µg/mL; lane 6, cefotaxime 0.24 µg/mL + 220 µg/mL CUR. Data correspond to mean values ± SD of three independent experiments carried out in triplicate. *** *p* < 0.001, and **** *p* < 0.0001.

Shiga toxin is the virulence factor that distinguishes the EHEC strain. The effect of CUR on STX gene expression was evaluated with RT-PCR. Gel analysis revealed that in the DMSO-treated cultures, *stx2A* and *stx2B* were overexpressed compared with cultures treated with CUR or antibiotics (Figure 5A,C). Interestingly, stx2A expression levels showed minor fold changes of 0.37 and 0.5 in CUR–chloramphenicol and CUR–cefotaxime, respectively, compared with antibiotics alone (Figure 5B). Similar results were observed with the expression of stx2B showing fold changes of 0.61 for CUR–chloramphenicol and 0.44 for CUR–cefotaxime (Figure 5D).

## 4. Discussion

Pathogenic enterohemorrhagic *Escherichia coli* (EHEC) is responsible for large outbreaks worldwide. Antibiotic therapy is generally avoided due to the risk of inducing hemolytic uremic syndrome (HUS). However, in cases where treatment is deemed necessary, a targeted approach that prevents the triggering of stx2-harboring phages or the increase in stx2 transcription and Shiga toxin 2 (Stx2) production may be a viable option [9]. The use of DNA-damaging antibiotics, such as fluoroquinolones, triggers the SOS response, a crucial regulator of Shiga toxin production and the dissemination of antibiotic resistance [27]. Other antibiotics, such as chloramphenicol, a protein synthesis inhibitor, or cefotaxime, a bacterial cell wall synthesis inhibitor, do not directly damage DNA. Furthermore, cefotaxime’s rapid bactericidal effect may help mitigate prolonged adaptive responses that could lead to toxin production [6,28].

On the other hand, CUR derived from the rhizome of *Curcuma longa* L. has gained attention due to its antimicrobial properties: it has been shown to be effective against both Gram-positive and Gram-negative bacteria to various extents, depending on the type of bacterium and its cell wall structure [29,30]. The present study demonstrates that CUR did not affect the growth of EHEC. In previous studies examining the activity of natural compounds in EHEC, it was reported that they induce significant damage, inhibit Stx2 secretion, and suppress biofilm formation [10,31,32]; in the case of CUR, its activity against *Escherichia coli* has been obtained when used in nanoformulations alone or combined with silver particles [33]. However, the specific effects of CUR on pathotypes require further study to provide a more definitive conclusion.

This study reveals high resistance of EHEC to cefotaxime, a third-generation cephalosporin, and chloramphenicol. These data are consistent with global trends among *E. coli* strains [34,35], underlining EHEC as a challenge that requires collective efforts. The potential of natural compounds, particularly CUR, to enhance the efficacy of antibiotics indicates a promising alternative. In *E. coli*, CUR has demonstrated significant potential in reducing resistance when combined with various antibiotics, thereby making resistant bacteria more susceptible [15,26]. This breakthrough offers hope for more effective treatment of bacterial infections. In addition, the drug-sensitizing activity of CUR in mammalian cells has been widely documented. In cancer treatment, it can increase the sensitivity of tumor cells to chemotherapies, thereby improving outcomes and reducing toxicity. It also enhances the effect of anti-inflammatory drugs, decreasing adverse effects [24,36,37]. Few works have explored CUR as a sensitizer in bacteria. Our findings reveal, for the first time, that CUR can act as a sensitizer of EHEC to chloramphenicol and cefotaxime treatment. This sensitizing effect is demonstrated by a significant increase in the size of the inhibition halo in the disk diffusion procedure, indicating the restored sensitivity of EHEC to these antibiotics. It has been reported that CUR controls antibiotic resistance by downregulating biofilm promotor genes such as *bap*, diminishing biofilm biomass [38]. Shivangi Yadav et al. reported a water-soluble curcumin derivative that inhibited the AcrAB-TolC efflux system [39]. Interestingly, in the present study, *bla_CTX-M-15_* and *catA* were remarkably downregulated by CUR treatment. These results back up evidence that CUR can help break resistance to antibiotics. In addition, a 0.01-fold decrease in *acrB* expression was observed with 330 µg/mL CUR, while both *acrA* and *tolC* were upregulated, showing 1.8- and 1.6-fold changes, respectively. These results are in agreement with Manoj Pun et al.’s, who demonstrated, in other bacteria, different expression patterns of the three components of the AcrAB-TolC efflux system in response to plant-derived phytochemicals [40]. *AcrB* is a major subunit of the AcrAB-TolC pump. In *E. coli*, the inhibition of AcrB has been related to a loss of function, and no compensatory activity from other efflux pumps has been described [41]. Further investigation is necessary to understand how CUR impacts the pump’s functionality.

Numerous studies have demonstrated that CUR exhibits a synergistic effect in combination with antibiotics against various bacterial strains [14,15]. Our research findings demonstrate that combining CUR and chloramphenicol or cefotaxime exerts bactericidal synergistic effects on resistant EHEC, suggesting that using these combinations could be a potential strategy against this strain. Combinations of chloramphenicol and plant metabolites such as carvacrol and thymol demonstrated the strongest anti-staphylococcal activity, suggesting that these metabolites enhance the activity of antibiotics by facilitating their access to targets or preventing resistance [42]. Moreover, cefotaxime combined with conessine, a steroidal alkaloid compound that inhibits efflux pumps, showed better potency against *Pseudomonas aeruginosa* [43]. The action mechanism of CUR in Gram-positive and Gram-negative bacteria is related to the cell wall composition; for example, in *E. coli*, it increases cell membrane permeability [44]. Our findings indicate that the potentiating impact of CUR on chloramphenicol and cefotaxime against resistant EHEC facilitates their access to targets. More studies are needed to understand the specific mechanism of action of these synergisms.

Shiga toxin (Stx), an AB5 exotoxin, is produced by two distinct bacterial genera: *Shigella dysenteriae* type 1 and Shiga toxin-producing *Escherichia coli* (STEC), including enterohemorrhagic *E. coli* (EHEC). It is the primary virulent factor in severe diseases such as hemorrhagic colitis. Our evidence shows the overexpression of *stx2A* and *stx2B* induced by dimethyl sulfoxide (DMSO), the diluent of CUR, which could potentially lead to a higher overall concentration of Shiga toxin, but this relationship is not always linear. In addition, no direct relation between DMSO and Shiga toxin has been reported. Interestingly, bacteria treated with CUR diluted in DMSO showed a 0.5 decrease in *stx* gene expression levels. In addition, we reported the downregulation of Shiga toxin induced by CUR for the first time, even in the presence of cefotaxime, with *Stx2A* showing a 0.51-fold change and *Stx2B* a 0.44-fold change, or chloramphenicol, with *Stx2A* showing a 0.37-fold change and *Stx2B* a 0.61-fold change. The cytoprotective effect of CUR against the activity of Shiga toxin on mammal cells has been previously described [45], but it is poorly understood in bacteria.

It would be interesting to analyze whether the CUR-mediated dysregulation of Shiga toxin expression is observed at the protein level, as this could provide relevant information on the mechanisms by which this compound exerts its modulatory effect on Stx2. However, further studies, including detailed proteomic analyses and complementary functional assays, are required to confirm this hypothesis and understand its biological implications.

## 5. Conclusions

According to the FDA, CUR is proven to be safe, and there has been no observed development of drug-resistant strains in response to its use. The enhanced therapeutic efficacy observed in this study highlights the potential of CUR in combating antibiotic resistance and reversing EHEC resistance. Further research is crucial to understanding the mechanisms of CUR and assessing its clinical suitability.

## Figures and Tables

**Table 1:** PCR primers and amplification conditions.

Gene	Sequence (5′ to 3′)	Amplification Conditions	Size (bp)
*bla_CTX-M-15_*	F: 5′-ATC CGA TTG CGG AAA AGC ACG TCA-3′ R: 5′-GGA ACG TTT CGT CTC CCA GCT-3′	95 °C for 60 s and 95 °C for 30 s, followed by 40 cycles at 66 °C for 30 s, 72 °C for 60 s, and 72 °C for 7 min	165
*catA1*	F: 5′-GGT GAT ATG GGA TAG TGT TCA CCC-3′ R: 5′-CTG AAT CGC CAG CGG CAT CA-3′	95 °C for 60 s and 95 °C for 30 s, followed by 40 cycles at 62 °C for 30 s, 72 °C for 60 s, and 72 °C for 7 min	325
*acrA*	F: 5′-ACA GAG GGT TTA CGC CTC TGG C-3′ R: 5′-TCT GCG ATC CGG TAG GCA CT-3′		190
*acrB*	F: 5′-GCG ATC CTC AAA CTG CCG GT-3′ R: 5′-TGC GTC ATG GTG CCA TCG GT-3′		377
*tolC*	F: 5′-ATC GGC CTG AGC CTT TCT GGG-3′ R: 5′-GTT AAC GCA CGC CAT TTC GAC-3′		296
*gapdh*	F: 5′-GGT TTT GGC CGT ATC GGT CGC A-3′ R: 5′-TGA ACG GTG GTC ATC AGA CCT TCG-3′		506
*stx2A*	F: 5′-ACG GTT TCC ATG ACA ACG GAC AGC-3′ R: 5′-AGA ACT GCT CTG GAT GCA TCT CTG G-3′	95 °C for 60 s and 95 °C for 30 s, followed by 40 cycles at 60 °C for 30 s, 72 °C for 60 s, and 72 °C for 7 min	164
*stx2B*	F: 5′-TGC AAT GGC GGC GGA TTG TGC-3′ R: 5′-ACT GCA CTT CAGCAA ATC CGG AGC-3	95 °C for 60 s and 95 °C for 30 s, followed by 40 cycles at 65 °C for 30 s, 72 °C for 60 s, and 72 °C for 7 min	209

**Table 2:** Antibiotic susceptibility pattern of EHEC.

Antibiotic	Inhibition Diameter (mm)
	EHEC
Cefotaxime (CTX)	25 ± 0 (I)
Chloramphenicol (C)	11 ± 0 (R)

**Table 3:** Antibiotic susceptibility pattern of EHEC previously sensitized with CUR.

CUR-Sensitized EHEC					
Antibiotic	Inhibition Diameter (mm)				
	Untreated	1.2% DMSO	110 µg/mL CUR	220 µg/mL CUR	330 µg/mL CUR
Cefotaxime (CTX)	25 ± 0 (I)	25 ± 0.7 (I)	31 ± 0 (S)	30 ± 1.4 (S)	33 ± 1.4 (S)
Chloramphenicol (C)	11 ± 0 (R)	12 ± 0 (R)	20 ± 0.7 (S)	21 ± 1.4 (S)	21 ± 1.4 (S)

**Table 4:** The antibiotic susceptibility pattern of EHEC in the presence of CUR.

0.24 μg/mL CTX + 1.2% DMSO	0.24 μg/mL CTX + 110 µg/mL CUR	0.24 μg/mL CTX + 220 µg/mL CUR	0.24 μg/mL CTX + 330 µg/mL CUR
25 ± 0.7 (I)	33 ± 0.7 (S)	34 ± 0.7 (S)	34 ± 0.7 (S)
64 μg/mL C + 1.2% DMSO	64 μg/mL C + 110 µg/mL CUR	64 μg/mL C + 220 µg/mL CUR	64 μg/mL C + 330 µg/mL CUR
12 ± 0 (R)	14 ± 0.7 (I)	15 ± 1.4 (I)	15 ± 1.4 (I)

CTX—cefotaxime; C—chloramphenicol.

**Table 5:** FIC indexes of CUR combinations with cefotaxime and chloramphenicol.

0.24 μg/mL CTX + 110 µg/mL CUR	0.24 μg/mL CTX + 220 µg/mL CUR	0.24 μg/mL CTX + 330 µg/mL CUR
FIC index of 0.86	FIC index of 0.62	FIC index of 0.01
64 μg/mL C + 110 µg/mL CUR	64 μg/mL C + 220 µg/mL CUR	64 μg/mL C + 330 µg/mL CUR
FIC index of 1.30	FIC index of 0.93	FIC index of 0.46

## Data Availability

The data presented in this study are available in this article.
